# Safety, efficiency and health-related quality of telephone triage conducted by general practitioners, nurses, or physicians in out-of-hours primary care: a quasi-experimental study using the Assessment of Quality in Telephone Triage (AQTT) to assess audio-recorded telephone calls

**DOI:** 10.1186/s12875-020-01122-z

**Published:** 2020-05-09

**Authors:** D. S. Graversen, M. B. Christensen, A. F. Pedersen, A. H. Carlsen, F. Bro, H. C. Christensen, C. H. Vestergaard, L. Huibers

**Affiliations:** 1 Research Unit for General Practice, Aarhus, Bartholins Allé 2, 8000 Aarhus C, Denmark; 2 Department of Public Health, Aarhus University, Bartholins Allé 2, 8000 Aarhus C, Denmark; 3 Department of Clinical Medicine, Aarhus University, Aarhus, Denmark; 4 Emergency Medical Services, Copenhagen, Denmark; 5 The National Clinical Databases (RKKP), Copenhagen, Denmark

**Keywords:** Triage, Telephone, After-hours care, Out-of-hours, Primary health care, General practitioners, Nurses, Safety, Efficiency, Quality of health care

## Abstract

**Background:**

To explore and compare safety, efficiency, and health-related quality of telephone triage in out-of-hours primary care (OOH-PC) services performed by general practitioners (GPs), nurses using a computerised decision support system (CDSS), or physicians with different medical specialities.

**Methods:**

Natural quasi-experimental cross-sectional study conducted in November and December 2016. We randomly selected 1294 audio-recorded telephone triage calls from two Danish OOH-PC services triaged by GPs (*n* = 423), nurses using CDSS (*n* = 430), or physicians with different medical specialities (*n* = 441). An assessment panel of 24 physicians used a validated assessment tool (Assessment of Quality in Telephone Triage - AQTT) to assess all telephone triage calls and measured health-related quality, safety, and efficiency of triage.

**Results:**

The relative risk (RR) of *poor* quality was significantly lower for nurses compared to GPs in four out of ten items regarding identifying and uncovering of problems. For most items, the quality tended to be lowest for physicians with different medical specialities. Compared to calls triaged by GPs (reference), the risk of *clinically relevant* undertriage was significantly lower for nurses, while physicians with different medical specialties had a similar risk (GP: 7.3%, nurse: 3.7%, physician: 6.1%). The risk of *clinically relevant* overtriage was significantly higher for nurses (9.1%) and physicians with different medical specialities (8.2%) compared to GPs (4.3%). GPs had significantly shorter calls (mean: 2 min 57 s, SD: 105 s) than nurses (mean: 4 min 44 s, SD: 168 s).

**Conclusions:**

Our explorative study indicated that nurses using CDSS performed better than GPs in telephone triage on a large number of health-related items, had a lower level of clinically relevant undertriage, but were perceived less efficient. Calls triaged by physicians with different medical specialities were perceived less safe and less efficient compared to GPs. Differences in the organisation of telephone triage may influence the distribution of workload in primary and secondary OOH services. Future research could compare the long-term outcomes following a telephone call to OOH-PC related to safety and efficiency.

## Background

The pressure on the out-of-hours (OOH) healthcare services, i.e. OOH primary care (OOH-PC), emergency departments (EDs), and prehospital emergency medical services (EMS), is increasing in many countries [1, 2]. Telephone triage plays a pivotal role in managing patient flows and workload [1, 3, 4]. Securing a safe and efficient telephone triage is a challenge as it must balance a minimum of undertriage securing high patient safety, while keeping overtriage at an acceptably low level. Existing OOH-PC services vary and use different triage models [5, 6], and involvement of general practitioners (GPs) is debated [1, 4, 7]. Many countries experience increasing shortage of GPs [8, 9], and GPs report high self-perceived stress and multiple burnout symptoms [10, 11]. In most countries, telephone triage in OOH-PC services is performed by nurses using a computerised decision support system (CDSS) [6]. In Denmark, GPs primarily perform the telephone triage [3].

Previous studies have explored the safety and efficiency of telephone triage in OOH-PC services [12–23]. Some have questioned the safety of telephone triage conducted by nurses [13, 24], especially for high-risk calls [24]. Newer studies suggest nurse triage to be safe [1, 12, 20, 25], and concerns mainly regard efficiency [1, 26]. However, most previous studies have described only nurse-led telephone triage conducted in study settings using vignettes [17, 18], simulated patients [13, 15, 19], or review of patient records [20]. This approach has provided little uniformity of outcome measures regarding accuracy of triage, patient safety, and efficiency [16]. Moreover, comparative studies of nurse- and GP-led telephone triage are sparse and mostly describe the quality of telephone triage in daytime [25, 27] rather than OOH [12]. To our knowledge, no existing studies have compared telephone triage by physicians with different specialities. Consequently, comparative studies of the quality of OOH telephone triage by GPs, nurses, and physician with different specialities in natural settings with real patient calls are needed.

After a reorganisation in 2014, two organisations for OOH-PC exist in Denmark alongside, one with nurse-led telephone triage using CDSS and physician-led triage, and one with GP-led telephone triage. This situation made it possible to explore the quality of the two OOH telephone triage models in a natural setting. In this study, we aim to explore and compare the safety, efficiency, and health-related quality of telephone triage at OOH-PC services performed by GPs, nurses using CDSS, or physicians with different medical specialities.

## Methods

### Design and setting

We conducted a natural quasi-experimental study in two OOH-PC services in Denmark. We selected the GPC in the Central Denmark Region using GP-led telephone triage and the medical helpline 1813 (MH-1813) in the Capital Region of Denmark using telephone triage performed by registered nurses with a CDSS and physicians with different medical specialities (see Table 1).

**Table 1:** Description of the OOH organisations in two included telephone triage models

	GP cooperative (GPC)	Medical helpline 1813 (MH-1813)
**Region**	**Central Denmark Region**	**Capital Region of Denmark**
**Population**	1.2 m citizens [28]	1.8 m citizens [29]
**Telephone calls in 2014** [30]	697,000	911,000
**Organiser**	GPs in the region	Regional administration
**Organisation and services**	▪ Telephone triage, home visits, and face-to-face consultations at the GPC ▪ GPs are obliged to take part in the service	▪ Telephone triage and home visits run by MH-1813 ▪ Face-to-face consultations are located in hospital facilities and managed by EDs
**Remuneration of professionals**	Fee for service	Payment by the hour
**Triage professional**	GPs or GP trainees in their final year of speciality; no CDSS available	Nurses who are obliged to use a CDSS and option to redirect calls to a physician Physicians with different medical specialities (a minority being GPs)

In 1992 a reform introduced large-scale GPCs, with GP specialists performing telephone triage [3]. In the Capital Region of Denmark a reorganisation in January 2014 formed the MH-1813, where nurses answer calls using a CDSS with the option to redirect calls to physicians on duty. All triage nurses are certified as registered nurses indicating a completed 3.5-year professional bachelor’s degree and completed a 6-week introductory course when employed in MH-1813, and MH-1813 conducts regular audits of nurse calls. Besides answering the redirected calls from nurses, physicians answer approximately one third of all calls to the MH-1813 directly. Physicians employed at MH-1813 have different medical specialties (e.g. internal medicine, pediatrics, anesthesiology, surgery) and varying experience (including junior physicians), with only a minority being a GP. We refer to this group as *physicians* in the rest of the article. The CDSS is also accessible for physicians, without an obligation to use it [Personal communication with MH-1813] [31]. The GPC and the MH-1813 are open outside office hours, i.e. on weekdays from 4 pm to 8 am, weekends all day, and national holidays, offering telephone consultations, clinic consultations and home visits. The MH-1813 is accessible 24 h/ per day, but only calls outside office hours were included to match the GPC opening hours. The OOH-PC services routinely audio-record all calls and have an administrative registration system. We were unable to access patient information on ethnicity, educational level, socio-economic status, or comorbidity.

### Selection of calls

**Fig. 1:**
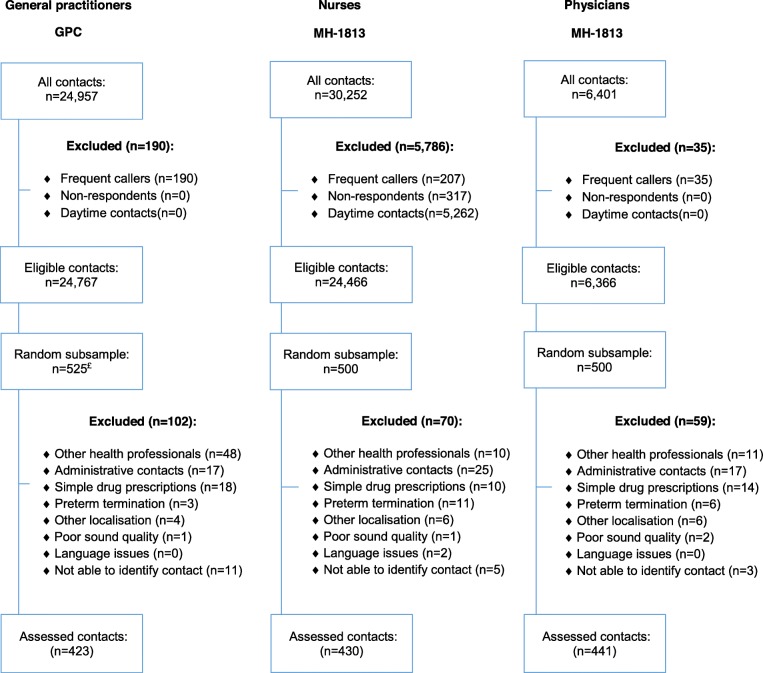
Flowchart of selection and exclusion of calls from the GPC and MH-1813. Note: For definition of exclusion criteria see Table 2; ^£^More calls were selected from the GPC, to account for the higher expected number of calls from other health professionals

We aimed to include an equal distribution of calls triaged by GPs, nurses, and physicians. For our power calculation, we used the level of undertriage, as this potentially has most clinical implications. Based on literature, we assumed an undertriage rate of approximately 9.5% for a power calculation, an ability to detect a 5% difference in undertriage between triage professionals, with a power of 0.8 and an alpha of 0.05. Thus, 435 calls per group of triage professionals were needed. All calls answered directly by a triage professional at GPC or MH-1813 outside office hours during the inclusion period (MH-1813: 23 November − 8 December 2016, GPC: 23 November - 7 December 2016) were eligible (Fig. 1). For calls redirected by a nurse to a physician at MH-1813, only the part conducted by the nurse was eligible. Based on available registration information, we selected eligible calls (Table 2). From all eligible calls we randomly selected (500 calls per triage professional group, matching the overall distribution on day of week (i.e. weekend/not weekend) and time of day (i.e. day, evening, night) using STATA. We selected 525 GPC calls, as we expected more exclusions due to the lack of a separate direct telephone number for nursing homes. Each selected call had a unique identification number that was used to identify the corresponding audio-recorded call.

**Table 2:** Exclusion criteria

Type	Definition/clarification
Frequent callers	Defined as patients with ≥7 calls during the two-week inclusion period (assessment of the triage quality could be difficult as the patient’s medical record from the OOH service could include important information on these patients that was available only to the triage professional and not to the assessor)
Call by mistake	Calls with no caller answering the triage professional.
Daytime calls	Calls performed during daytime (the telephone triage service at MH-1813 was available during daytime)
Other health professionals	The caller was another healthcare professional, e.g. from a nursing home
Administrative calls	The reason for calling was administrative, e.g. calling to get the number for the acute dentist
Simple drug prescriptions	The patient called for renewal of a prescription that required little information sharing
Preterm termination	Calls that were ended too early, e.g. calls made by error, no sound on call, or sound interrupted in the middle of call
Other localisation	Calls from a caller who was not in the same location as the patient, e.g. parent on the way to pick up a sick child from day care
Poor sound quality	Calls with poor sound quality (making assessment difficult)
Language issues	Calls in which language issues challenged the triage, i.e. caller did not speak Danish or English
Not able to identify call	Random calls where an exact linkage to the corresponding audio-recorded call or the audio recording could not be established

Three master students of medicine masked the audio recordings using beep tones to mask triage profession, OOH organisation, and patient identification information. These medical students were trained and each student was supervised for the first 20 calls by DSG in the masking and exclusion process. If a call fulfilled or if the student was in doubt if the call fulfilled the exclusion criteria. Final decisions to exclude or not were made by first author (DSG), or if in doubt, a consensus was reached between DSG and AFP. Due to an unforeseen partly system failure of the IT system at MH-1813 for 3 days, we were unable to get the audio-recordings of 194 selected calls (22% of all MH-1813 calls). We substituted these with randomly selected calls from the following week, matching on day of week and time of day.

### Assessment tool

Assessments were performed using the tool “Assessment of Quality in Telephone Triage” (AQTT) and the accompanying rating manual printed in a booklet (Appendices 1 and 2 provides an overview of the 24 items and the general rating scale for most specific items). The AQTT was thoroughly developed and tested, with satisfactory inter-rater agreement when distinguishing *poor* from *sufficient* performance [32]. The AQTT comprises 24 items assessing the health-related quality (eleven specific items), quality of communication (nine specific items), as well as four overall items of the assessors’ general perception of the quality of communication, health-professional quality, patient safety, and efficiency. The majority of items are rated on a 5-point Likert scale with an additional category “not applicable” (“n/a”) if an item is correctly found not relevant or available information is insufficient for assessment. The accuracy of the triage decision (item 11) is assessed on a 7-point scale to differentiate between levels of undertriage and overtriage (defined in footnotes of Table 6). The AQTT provides explicit definitions of when to apply the specific ratings for each item, including when to score “n/a”. Overall items are measured on a 10-point visual analogue scale, representing the general perception of the assessor, after scoring of all specific items. We present results on the eleven health-related items and three overall items (Table 3).

**Table 3:** Overview of specific health- professional items and items assessing overall quality

**Items assessing specific health-related aspects**	
1:	Collects information about location
2:	Asks to speak to the patient when caller has briefly described the situation
3:	Identifies and acts appropriately on signs that could be critical or life-threatening for the patient (signs of problems according to the ABCDE criteria)
4:	Identifies and uncovers problems, including symptoms and their development
5:	Identifies and states the purpose of the patient’s call
6:	Prioritises the presented problems and symptoms appropriately
7:	Asks (as a minimum) all essential questions concerning the problem(s) and symptom(s) to gain the information required for optimal triage
8:	Asks the relevant questions concerning previous medical history and medications
9:	Gives relevant advice on self-care
10:	Gives relevant advice on safety netting
11:	Selects optimal triage decision
**Items assessing overall quality:**	
22:	How would you assess the overall health-professional quality?
23:	How would you assess the overall patient safety?
24:	How would you assess the overall efficiency?

Items 12 to 21 focused on the quality of communication, which will be presented in another paper

### Assessment panel

We recruited 24 physicians for the assessment panel among triage professionals from the GPC and MH-1813 using two inclusion criteria: > 1 year experience and currently active in telephone triage in OOH-PC. An email invitation was sent to all GPs and physicians by their organisers. Using STATA we randomly selected 16 GPs from the 56 interested GPs at the GPC, matching age and sex distribution. At the MH-1813, we included all eight physicians fulfilling our inclusion criteria from the ten interested physicians. All assessors followed a two-day training course providing knowledge on telephone triage and communication, introducing the AQTT and rating manual, and assessing triage calls individually and in plenary, focusing on achieving consistency.

### Assessment process

After collection, we renamed all audio-recorded calls and distributed them at random to the assessment panel, regardless of OOH service, with one assessor per call. Thus, each assessor assessed calls by all triage professionals. Information on age and sex of the patient, day of week, and the time of each call was available. Assessors made their assessments at home; each assessed a median of 53 (range: 48 to 61) calls during a median period of 111 days.

### Statistical analyses

For health-related specific items, we categorized the outcomes into *poor* quality (rated “1” or “2”) and *sufficient* quality (rated “3”, “4”, or “5”). “Not applicable” (“n/a”) was recoded into “missing”. Accuracy of triage decision (item 11) was categorised into *clinically relevant* undertriage (rated “1” or “2”) and *clinically relevant* overtriage (rated “6” or “7”). These categorizations were based on the satisfactory inter-rater agreement of the AQTT [32].

We used descriptive analyses to describe patient and call characteristics stratified by triage professional group. We conducted an overall comparison of patient and call characteristics using chi-square test for categorical variables and Kruskal-Wallis test for continuous variables (significance level < 0.05). In case of a significant difference, we conducted a post-hoc pair-wise comparison using chi-squared test for categorical variables and Mann-Whitney U-test for continuous variables with Bonferroni adjusted significance level (< 0.025). We also used descriptive analysis to describe the ordinal-scaled health-related specific items, excluding the rating “n/a” from our analyses. We calculated the relative risk (RR) of having *poor* quality (i.e. rated “1” or “2”) versus *sufficient* quality (i.e. rated “3”, “4” or “5”*)* on the health-related specific items and of *clinically relevant* undertriage or overtriage (vs. not *clinically* relevant undertriage or overtriage) for the three groups of triage professionals, using binomial regression. All comparative analyses were conducted pairwise using GP-led triage as reference group. The items measuring the overall perceived quality were compared by ranksum between triage professional using non-parametric Mann-Whitney U-test as most distributions did not follow normal distribution.

We noticed a tendency to overestimate the quality of GP-led triage for assessors from GPC (i.e. GPs) compared with assessors from MH-1813 as well as the reverse: assessors from MH-1813 overestimating the quality of physician-led triage compared with GP assessors. We concluded that a “similar-to-me” bias was present in the data, i.e. assessors giving a slight bonus to triage led by a similar triage professional to themselves [33]. Since the dataset is unbalanced (GPC: 16 vs. MH-1813: 8) and, more importantly, since nurses could never receive such favorable assessment, we decided to adjust the RR estimates of *poor quality* and of *clinically relevant* under- and overtriage for whether or not assessor had the same professional background as the triage professional. All analyses were performed in STATA 14.2 (StataCorp. 2015. *Stata Statistical Software: Release 14.2*. College Station, TX: StataCorp LP).

## Results

### Population

In our final analyses, we included 423 calls triaged by GPs, 430 by nurses, and 441 by physicians of different medical specialties (Fig. 1). No differences in triage calls were identified between GPs and nurses and between GPs and physicians concerning patients’ age and sex and time of call (Table 4). An explorative analysis comparing calls of nurses and physicians revealed a significant difference in patients’ sex (*p* = 0.006 not shown in table). Nurse telephone calls were significantly longer (mean = 4 min 44 s, SD: 168 s) compared to calls triaged by GPs (mean = 2 min 57 s, SD 105 s) and physicians (mean = 4 min 1 s, SD: 146 s).

**Table 4:** Baseline distribution of patient and call characteristics, stratified by triage professional group

Triage professional	GP (***n*** = 423)	Nurse (***n*** = 430)	Physician (***n*** = 441)
**Patient characteristics**			
Sex, % (n) ^£^			
Male	42.8 (181)	37.9 (163)	47.2 (208)
Female	57.2 (242)	62.1 (267)	52.8 (233)
Age group in years, % (n)			
0–4	20.3 (86)	23.6 (101)	21.9 (96)
5–17	15.8 (67)	13.3 (57)	14.8 (65)
18–39	29.6 (125)	31.5 (135)	30.6 (134)
40–64	21.8 (92)	20.6 (88)	20.1 (88)
≥65	12.5 (53)	11.0 (47)	12.6 (55)
**Call characteristics**			
Time of call^a^, % (n)			
Weekend	51.6 (218)	51.2 (220)	50.3 (222)
Not weekend	48.5 (205)	48.8 (210)	49.7 (219
Day	22.2 (94)	22.6 (97)	21.1 (93)
Evening	61.5 (260)	60.9 (262)	61.5 (271)
Night	16.3 (69)	16.5 (71)	17.5 (77)
Length of call, min and sec (SD - sec)^£^			
Mean	2 min 57 s (105) ^*b*^	4 min 44 s (168)*	4 min 1 s (146)*

£ Indicating a significant difference (*p* < 0.05) between all three groups of triage professionals, using chi-square test for categorical variables and Kruskal-Wallis for length of call

*Significant difference between nurses or physicians in pairwise comparison with GPs as reference group (Bonferroni adjusted *p* < 0.025), using chi-squared test (all categorical variables) and Mann-Whitney U-test (length of call)

^a^Time of call: Weekend = Friday 4 pm - Sunday midnight; Not weekend = Monday 0 am - Friday 8 am; ^b^Available only for 352 of 423 calls from GPC

### Health-related specific items

**Fig. 2:**
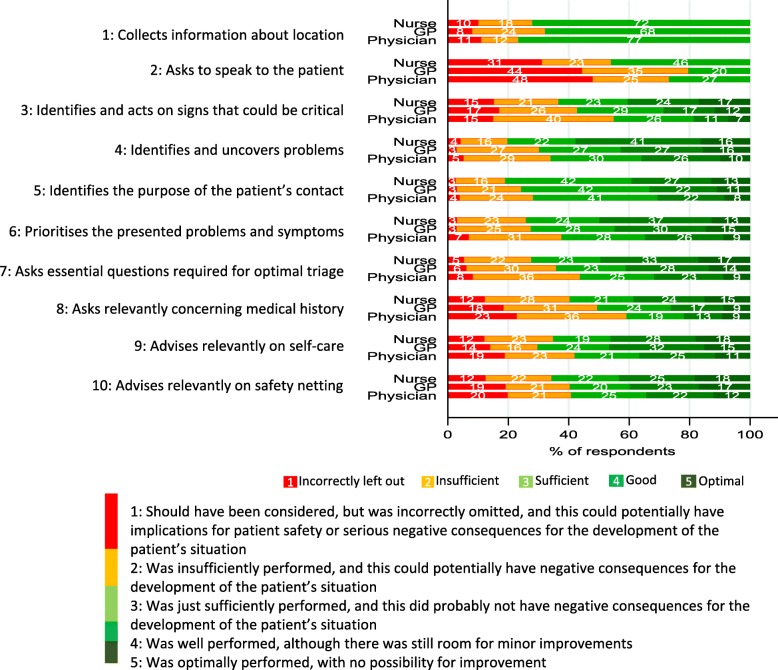
Distribution of assessments when item was applicable*.* Note: Distribution of ratings for each specific health-related item. When an item was scored as “not applicable”, the call was excluded from the distribution for that particular item. Items 1 and 2: The scale for items 1 and 2 ranges from only one to three, as performance can only be insufficiently performed or performed but with no possibility to excel (thus, “good” or “optimal” performance is not possible). Item headlines in abbreviated form. For full length headlines, see Table 4

Figure 2 shows the distribution of ratings for each group of triage professionals, with varying use of “n/a” between items and between triage professional. For four items the RR of *poor* quality was significantly lower for nurses compared with GPs: “asks to speak to patient” (RR = 0.68, 95% CI: 0.52–0.89), “identifies problems” (RR = 0.66, 95% CI: 0.52–0.83), “asks essential questions” (RR = 0.77, 95% CI: 0.63–0.94), and “asks about medical history” (RR = 0.82, 95% CI: 0.68–0.97) (Table 5). Physicians had a significantly higher RR of a *poor* quality than GPs for four items (i.e. 6, 7, 8, 9). Table 5 additionally, shows the RR estimates adjusted for evaluator background (GPC, MH-1813) (i.e. similar-to-me) and the uneven constitution of assessors (assessors from GPC:MH-1813 – 16:8).

**Table 5:** Assessment of percentage poor and relative risk (RR) of poor quality of health-related items for different triage professionals

Health-related specific items (AQTT)	Triage professional	Not applicable^**a**^ (%)	Poor quality % (n)	RR for poor quality (95% CI)	Adjusted RR^**c**^ poor quality (95% CI)
**1: Collects information about location**^**b**^	GP	70.7	32.3 (40)	1	1
	Nurse	60.9	28.0 (47)	0.87 (0.61–1.23)^*P* = 0.43^	0.91 (0.61–1.34) ^*P* = 0.62^
	Physician	65.1	23.4 (36)	0.72 (0.49–1.01) ^*P* = 0.10^	0.75 (0.51–1.10) ^*P* = 0.142^
**2: Asks to speak to the patient when the caller has briefly described the situation**^**b**^	GP	87.2	79.6 (43)	1	1
	Nurse	85.8	54.1 (33)	0.68 (0.52–0.89)* ^*P* = 0.01^	0.71 (0.51–0.98)* ^*P* = 0.04^
	Physician	83.9	73.2 (52)	0.92 (0.76–1.12) ^*P* = 0.40^	0.94 (0.75–1.17) ^*P* = 0.57^
**3: Identifies and acts appropriately on signs that could be critical or life-threatening for the patient (signs of problems according to the ABCDE criteria)**	GP	73.5	42.9 (48)	1	1
	Nurse	69.5	36.6 (48)	0.85 (0.63–1.17) ^*P* = 0.32^	0.74 (0.55–1.00) ^P = 0.05^
	Physician	68.3	55.0 (77)	1.28 (0.99–1.67) ^*P* = 0.06^	1.31 (1.00–1.70)* ^*P* = 0.05^
**4: Identifies and uncovers problems, including symptoms and their development**	GP	1.0	30.3 (127)	1	1
	Nurse	0.5	19.9 (85)	0.66 (0.52–0.83)* ^*P* = 0.00^	0.61 (0.47–0.80)* ^*P* = 0.00^
	Physician	0.2	34.1 (150)	1.12 (0.93–1.37) ^*P* = 0.24^	1.09 (0.89–1.34) ^*P* = 0.39^
**5: Identifies and states the purpose of the patient’s call**	GP	20.3	24.3 (82)	1	1
	Nurse	19.3	19.0 (66)	0.78 (0.59–1.04) ^*P* = 0.09^	0.76 (0.54–1.70) ^*P* = 0.12^
	Physician	19.1	28.3 (101)	1.16 (0.91–1.49) ^*P* = 0.24^	1.14 (0.86–1.50) ^*P* = 0.37^
**6: Prioritises the presented problems and symptoms in an appropriate way**	GP	1.2	27.5 (115)	1	1
	Nurse	0.5	25.9 (111)	0.94 (0.75–1.18) ^*P* = 0.0.60^	0.81 (0.63–1.03) ^*P* = 0.8^
	Physician	1.8	37.6 (163)	1.37 (1.12–1.67)* ^*P* = 0.00^	1.28 (1.05–1.57)* ^*P* = 0.02^
**7: Asks, as a minimum, all the essential questions concerning the problem(s) and symptom(s) required for optimal triage**	GP	0.5	35.9 (151)	1	1
	Nurse	0.0	27.7 (119)	0.77 (0.63–0.94)* ^*P* = 0.01^	0.74 (0.59–0.93)* ^*P* = 0.01^
	Physician	1.1	43.8 (191)	1.22 (1.03–1.44)* ^*P* = 0.02^	1.20 (1.01–1.42)* ^*P* = 0.04^
**8: Asks the relevant questions concerning previous medical history and medications**	GP	32.2	49.5 (142)	1	1
	Nurse	24.0	40.4 (132)	0.82 (0.68–0.97)* ^*P* = 0.02^	0.75 (0.61–0.91)* ^*P* = 0.00^
	Physician	28.3	59.2 (187)	1.20 (1.03–1.39)* ^*P* = 0.02^	1.15 (0.98–1.34) ^*P* = 0.09^
**9: Gives relevant advice on self-care**	GP	34.0	29.8 (83)	1	1
	Nurse	52.1	35.0 (72)	1.17 (0.91–1.52) ^*P* = 0.22^	0.93 (0.71–1.22) ^*P* = 0.60^
	Physician	38.6	42.1 (114)	1.41 (1.13–1.78)* ^*P* = 0.00^	1.30 (1.03–1.64)* ^*P* = 0.03^
**10: Gives relevant advice on safety netting**	GP	36.9	40.5 (108)	1	1
	Nurse	55.4	34.4 (66)	0.85 (0.67–1.08) ^*P* = 0.20^	0.75 (0.58–0.97)* ^*P* = 0.03^
	Physician	41.7	40.9 (105)	1.01 (0.82–1.24) ^*P* = 0.93^	0.98 (0.79–1.20) ^*P* = 0.81^

The RR for “poor quality” (i.e. “1” or “2”) was analysed using binomial regression model (GP as reference group). *Significant differences: *p* < 0.05

^a^Not applicable was expected in a considerable proportion of cases, in line with the instructions for assessment in the guideline (see methods). We calculated the percentage of calls with “poor quality” (i.e. rated “1” or “2”) of all calls in which the item was relevant (i.e. “not applicable” excluded). ^b^Items 1 and 2 were rated from “1” to “3”;

^c^ RR of poor quality adjusted for evaluator background (GPC, MH-1813) (i.e. if call is assessed by an assessor with the same professional background and organisation (similar-to-me)) and the uneven constitution of assessors (ratio assessors from GPC:MH-1813 – 16:8)

### Accuracy of triage outcome

Only 3.7% of calls triaged by nurses were *clinically relevan*t undertriaged, whereas GPs (7.3%) and physicians (6.1%) had higher percentages (Table 6). Consequently, the risk of *clinically relevant* undertriage was significantly lower for nurses compared to GPs (RR = 0.51, 95% CI: 0.28–0.93). Compared to GP-led triage, the risk of being *clinically relevant* overtriaged was significantly higher for nurse-led (RR = 2.13, 95% CI: 1.22–3.73) and physician-led triage (RR = 1.93, 95% CI: 1.10–3.39).

**Table 6:**
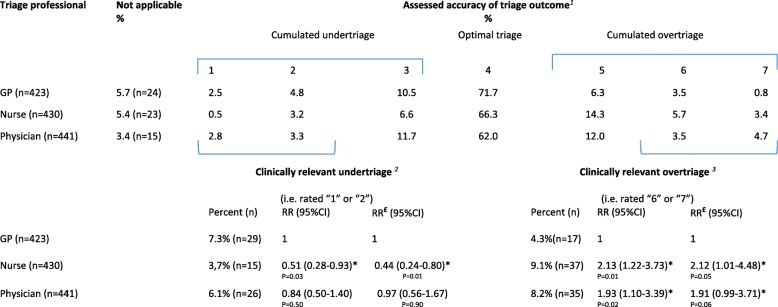
Assessed triage decision and relative risk (RR) of optimal triage, undertriage and overtriage for triage professionals

^1^Rating scale assessing appropriateness of triage decision with definitions of each rating: 1. Severe undertriage: The call is undertriaged with risk of severe consequences; 2. Moderate undertriage: The call is undertriaged, but unlikely with risk of severe consequences; 3. Mild undertriage: The call is undertriaged, but could have been triaged “somewhat higher”; 4. Optimal triage: The call is optimal triaged; 5. Mild overtriage: The call is overtriaged, but could have been triaged “somewhat lower”; 6. Moderate overtriage: The call is overtriaged, it would have been sufficient with a “less burdensome service”; 7. Severe overtriage: The call is overtriaged; it seems completely irrelevant to choose this triage outcome

^2^Clinically relevant undertriage is the sum of ratings “1” and “2”; ^3^Clinically relevant overtriage is the sum of “6” and “7”; The RR for “clinically relevant undertriage” and clinically relevant overtriage was analysed using binomial regression model. *Significant differences: *p* < 0.05

£ RR of poor quality adjusted for evaluator background (GPC, MH-1813) (i.e. if call is assessed by an assessor with the same professional background and organisation (similar-to-me)) and the uneven constitution of assessors (ratio assessors from GPC:MH-1813 – 16:8)

### Overall perceived quality

The overall perceived health-professional quality and efficiency of telephone triage was significantly lower for both nurses and physicians compared with GPs (Table 7). The overall perceived patient safety was significantly lower for physicians compared with GPs.

**Table 7:** Assessed overall health-related quality, safety, and efficiency per triage professional

Overall assessed quality (AQTT)^**a**^	Triage professional	Median (10th 90th percentile)
22: How would you rate the overall health-professional quality in the telephone triage?	GP	7 (3 to 10)
	Nurse	6 (2 to 9)* ^*P* = 0.00^
	Physician	6 (2 to 9)** ^*P* = 0.00^
23: How would you rate the overall patient safety in the telephone triage?	GP	8 (3 to 10)
	Nurse	8 (3 to 10) ^*P* = 0.09^
	Physician	7 (2 to 10)* ^*P* = 0.03^
24: How would you rate the overall efficiency in the telephone triage?	GP	8 (4 to 10)
	Nurse 1813	6 (2 to 9)** ^*P* = 0.00^
	Physician 1813	7 (2 to 10)** ^*P* = 0.00^

Median (10th 90th percentile): Quality was compared to GP-led triage by rank sum using Mann-Whitney U-test. Indicating a significant difference from GP triage, *p < 0.05, **p < 0.001

^a^Items were rated on a scale from 0 to 10 (0 = very low quality; 10 = optimal quality)

## Discussion

### Principal findings

We found a significant lower risk of *poor* quality for nurse triage compared to GP triage in four out of ten health-related items that focus on identifying and uncovering the problem and requesting to talk directly to the patient. In four out of ten items, the risk of *poor* quality was significantly higher in calls triaged by physicians with different medical specialities compared to GPs. The risk of *clinically relevant* undertriage was significantly lower for nurses compared to GPs. However, compared to GPs, both nurses and physicians had significantly more *clinically relevant* overtriage. In addition, the calls were significantly longer for nurses compared to GPs, and the overall perceived efficiency was significantly higher in GP-led telephone triage compared to nurse-led and physician-led triage. The overall perceived safety was significantly lower in physician-led triage and tended to be higher in nurse-led triage compared to GP-led triage.

### Strengths and weaknesses of the study

To our knowledge, this is the first study to compare the quality of OOH telephone triage performed by GPs, nurses using CDSS, and physicians in a real-life setting. Major strengths are the use of randomly selected real-life calls as opposed to the constructed setup used in previous studies [18–20, 34, 35] and the assessment of a range of outcome measures. Additional strengths are the study size with 1294 calls and the meticulous assessment process using the validated AQTT tool combined with a comprehensive rating manual that included clear definitions per answering category for each item, thus reducing the subjectivity of the assessments.

Our study also had some limitations. Multiple assessors per call would have been preferable, but due to the thorough assessment process, this was not feasible. Thus, each call was only assessed by a single assessor. We took several precautions to ensure consistency of assessments; the assessors followed a comprehensive training course, assessments followed the carefully developed and validated AQTT [32], and audio-recordings were attempted masked for information about organisation and triage professional. Moreover, in comparative analyses we dichotomised ratings (distinguishing *poor* from *sufficient* quality), which was supported by the satisfactory inter-rater agreement of the AQTT [32].

Post-hoc sensitivity analyses revealed a similar-to-me cognitive bias [33], indicating that the risk of *poor* quality in calls assessed by an assessor similar to the triage professional tended to be assessed lower than if not assessed by a similar assessor. Furthermore, the decision to include only physicians (GPs from GPC and physicians from MH-1813) in the assessment panel may have induced cognitive bias when assessing nurse-led triage. We chose these assessors as no consensus exists on the best professional for assessing quality of telephone triage [13–15, 17, 36], and physicians or GPs have most frequently been used in other studies [13–15]. Moreover, our assessment panel was unbalanced with more assessors from the GPC compared to MH-1813 (16:8). We adjusted for the similar-to-me bias and for the uneven distribution of assessors. The adjusted RR of *poor* quality and of *clinically relevant* undertriage and overtriage generally favours nurse triage with lower RR of *poor* quality. The adjusted RR were comparable to the crude estimates but points towards smaller difference between GPs and physicians for most items. However, the use of non-parametric ranksum for the overall perceived quality items did not allow these adjustments. As these items encompass a high level of subjectivity, we assume that adjustment for these factors may have increased differences between the triage professionals.

No differences in calls were seen between the compared groups concerning age, sex, and time of call. We know that populations in the different regions differ, as the percentage of immigrants and the level of education is higher in the Capital Region (MH-1813) [28]. If these differences also exist for callers to the OOH services, this could potentially give case mix with different levels of difficulty in triage contacts. Moreover, data on other factors like co-morbidity and socioeconomic status were regrettably not available. In addition, some items had considerable proportions of “n/a” assessments, as intended, with significant differences between triage professionals in four items. Thus, some case mix cannot be rejected and should be considered especially when interpreting comparisons with small number of calls. Furthermore, we did not have access to background characteristics of the triage professionals, such as age, gender, experience, and education. The management of “n/a” was ambiguous as it could both reflect a correct performance (i.e. “*correctly found not relevant”)*, but could also potentially cover a *poor* performance (i.e. “*available information is insufficient for assessment*”). In the testing of the reliability of AQTT “n/a” was recoded into “3”, but for the purpose of this paper, we chose to exclude “n/a”. Managing “n/a” as “sufficient quality” could overestimate the quality. A post-hoc sensitivity analysis of the inter-rater ICC reliability excluding “n/a” did not change the reliability considerably, and always towards a higher reliability. In the analyses we have performed many tests so significance by change cannot be excluded. A solution could be adjusting significance levels by Bonferroni consistently throughout all analyses, but this has been suggested to be too conservative and associated with increased risk of type-2 errors [37].

### Interpretation and comparisons of results

Our study revealed that the quality of nurse-led triage using CDSS was higher than GP-led triage for most items and tended to be lower for physicians. However, we cannot say whether these differences are attributed to (non-)use of CDSS, differences in educational background, personality, and/or organisational conditions. CDSSs are developed to support health professionals in asking all essential questions [38] and ensuring consistency [39]. This corresponds to our finding that nurses are better at identifying and uncovering the problems. The differences between physicians and GPs, who did not use CDSS, could suggest that the medical background may be of relevance. The better ability of GPs to prioritise the problems and collect sufficient and complete information compared to physicians with different medical specialities could be attributed to GPs having more experience with similar unvisited patient populations in the daytime.

The rate of *cumulated* undertriage was 10.3% for nurses, 17.8% for GPs, and 17.8% for physicians, which is in line with other studies of nurse triage in controlled settings (12 to 41%) [13, 17, 18, 40]. To our knowledge undertriage has not been explored in GP triage. Two large-scale register-based randomised controlled trials comparing GP- and nurse-led telephone triage in daytime [25] and OOH [12] also suggested that nurse-led triage is safe, finding no excess deaths, hospital admissions, or increased ED attendance attributable to nurse-led triage.

Efficient OOH telephone triage incorporates multiple indicators, including overtriage and length of call. We found that the rate of *cumulated* overtriage was lowest in GP triage (GP: 11%, nurse: 23%, physician: 20%). The overtriage rate in other studies ranges from 12.5 to 19.3% in nurse-led triage [13, 17, 18]. Telephone calls triaged by nurses were significantly longer than calls triaged by GPs, which is supported by a study [41], but contradicted by another study [42]. The interpretation of the length of a call is ambiguous. A longer call may be more efficient if the problem is sufficiently resolved than a shorter call that does not sufficiently resolve the problem as this may lead to a new contact.

### Future research and practical implications

Our results show that decision-makers should be aware that different triage professionals can cause differences in the quality of telephone triage and may influence the distribution of workload in primary and secondary OOH services. Nurse-led triage as a solution for high GP workload seems feasible, but further research is needed in this field as fewer GPs are required in telephone triage but more GPs may be needed in face-to-face consultations.

Future research should compare the long-term outcomes following a telephone call to OOH primary care related to safety (e.g. mortality, hospital admission rates, and adverse events), efficiency (e.g. influence on GP workload, workload in the OOH services, and follow-up contacts), and patient satisfaction. Additionally, future research should investigate influence of using a CDSS and factors associated with potentially unsafe and inefficient calls, including the characteristics of the triage professional and the type of call.

## Conclusion

Keeping limitations in mind, our explorative study indicated that nurses using CDSS performed better than GPs in telephone triage, especially in four out of ten specific health-related items concerning identification and uncovering of the problem. Moreover, nurse-led triage was characterised by a lower level of *clinically relevant* undertriage, but more *clinically relevant* overtriage, and was perceived less efficient compared to GP-led triage. Calls triaged by physicians with different medical specialities were perceived less safe and less efficient compared to GPs and tended to receive lowest ratings on most specific items. The use of different triage professionals can influence the quality of telephone triage, and may influence the distribution of workload in primary and secondary OOH services. Future research could compare the long-term outcomes following a telephone call to OOH-PC related to safety and efficiency.

### Definitions

“Health-related quality”: the term health-related quality refer to the measured quality in the specific items (used in the red specific items in appendix 1).

“Health-professional quality”: the term health-professional quality refers to the measured quality exclusively in item 22 assessing the overall perceived health-professional quality.

## Supplementary information

**Additional file 1: Appendix 1**: Assessment tool, AQTT.

**Additional file 2: Appendix 2**: 5-point Likert rating scale of most specific items in AQTT.

## Data Availability

The anonymised data used and analysed during the current study are available from the corresponding author on reasonable request.

## Abbreviations

AQTT: Assessment of Quality in Telephone Triage (a validated tool for assessment of triage performance)
CDSS: Computerised decision support system
ED: Emergency departments
EMS: Emergency medical services
GP: General practitioners
GPC: GP cooperative
OOH: Out-of-hours
OOH-PC: Out-of-hours primary care
