# Supplementary material for: Safety, efficiency and health-related quality of telephone triage conducted by general practitioners, nurses, or physicians in out-of-hours primary care: a quasi-experimental study using the Assessment of Quality in Telephone Triage (AQTT) to assess audio-recorded telephone calls
Source: BMC Fam Pract. 2020 May 9;21:84. doi: 10.1186/s12875-020-01122-z (PMC7211335; doi:10.1186/s12875-020-01122-z)
Supplement: Supplementary file 1 — Additional file 1: Appendix 1: Assessment tool, AQTT. [file 12875_2020_1122_MOESM1_ESM.docx]

**Appendix 1: Assessment tool, AQTT**

| Communicative quality | |
| --- | --- |
| Health-related quality | |
| Overall quality | |
|  | |
| *INTRODUCTION (scale 1-3)* | |
| 1. | Collects information about location  (scale 1-3) |
| 2. | Asks to speak to the patient when the caller has briefly described the situation |
| *IDENTIFICATION AND UNCOVERING OF HEALTH PROBLEM (scale 1-5)* | |
| 3. | Identifies and acts appropriately on signs that could be critical or life-threatening for the patient (signs of problems according to the ABCDE criteria) |
| 4. | Identifies and uncovers problems, including symptoms and their development |
| 5. | Identifies and states the purpose of the patient’s call |
| 6. | Prioritises the presented problems and symptoms in an appropriate way |
| 7. | Asks, as a minimum, all the essential questions concerning the problem(s) and symptom(s) required  for optimal triage |
| 8. | Asks the relevant questions concerning previous medical history and medications |
| *TRIAGE* (scale 1-5) | |
| 9. | Gives relevant advice on self-care |
| 10. | Gives relevant advice on safety netting |
| 11. | Choses the optimal triage decision *(scale 1-7)* |
| *COMMUNICATION (scale 1-5)* | |
| 12. | Gives the caller sufficient time and space to describe the situation |
| 13. | The conversation is conducted in understandable language adapted to the caller’s situation |
| 14. | Ensures that the triage decision and the advice given are understandable and feasible |
| 15. | Ensures that the caller agrees on the triage decision and advice given and is accommodating in case of disagreement |
| 16. | Structures the conversation |
| 17. | Masters suitable questioning techniques (including suitable use of open-ended, closed-ended and non-leading questions) |
| 18. | Summarises (if relevant), verifies and adjusts if needed |
| 19. | Pays attention to the caller’s experience and situation |
| 20. | Conducts the conversation in an accommodating and friendly tone |
| *OVERALL QUALITY (scale 0-10)* | |
| 21. | How would you rate the overall communication in the telephone triage? |
| 22. | How would you rate the overall health-professional quality in the telephone triage? |
| 23. | How would you rate the overall patient safety in the telephone triage? |
| 24. | How would you rate the overall efficiency in the telephone triage? |
