# Supplementary material for: Safety, efficiency and health-related quality of telephone triage conducted by general practitioners, nurses, or physicians in out-of-hours primary care: a quasi-experimental study using the Assessment of Quality in Telephone Triage (AQTT) to assess audio-recorded telephone calls
Source: BMC Fam Pract. 2020 May 9;21:84. doi: 10.1186/s12875-020-01122-z (PMC7211335; doi:10.1186/s12875-020-01122-z)
Supplement: Supplementary file 2 — Additional file 2: Appendix 2: 5-point Likert rating scale of most specific items in AQTT. [file 12875_2020_1122_MOESM2_ESM.docx]

**Appendix 2:** 5-point Likert rating scale of most specific items in AQTT

| Not applicable | Only used if this aspect was correctly left out |
| --- | --- |
| Incorrectly left out (1) | Should have been considered, but was incorrectly omitted and this could potentially have implications for patient safety or serious negative consequences for the development of the patient’s situation |
| Insufficient (2) | Was insufficiently performed, and this could potentially have negative consequences for the development of the patient’s situation |
| Sufficient (3) | Was just sufficiently performed, and this did probably not have negative consequences for the development of the patient’s situation |
| Good (4) | Was well performed, although there was still room for minor improvements. |
| Optimal (5) | Was optimally performed, with no possibility for improvement. |
